# Supplementary material for: Physical drivers of the summer 2019 North Pacific marine heatwave
Source: Nat Commun. 2020 Apr 20;11:1903. doi: 10.1038/s41467-020-15820-w (PMC7171163; doi:10.1038/s41467-020-15820-w)
Supplement: Supplementary file 1 — Supplementary Information [file 41467_2020_15820_MOESM1_ESM.pdf]

Supplementary information for

Physical drivers of the summer 2019 North Pacific marine heatwave

by

Amaya[1,2] et al.

[1]Cooperative Institute for Research in Environmental Sciences, University of Colorado Boulder

[2]Department of Atmospheric and Oceanic Sciences, University of Colorado Boulder

Supplementary Methods:

Supplemental observational data

Monthly mean precipitation data was taken from the Global Precipitation Climatology Project (GPCP), which is available from 1979 to present on a 2.5˚x2.5˚ grid. GPCP integrates various satellite data sets over land and ocean, as well as precipitation gauge data over land. Anomalies using this data set are relative to a long-term monthly mean climatology for the period 1982-2018.

Low-cloud fraction was calculated from Level 3 satellite-derived monthly means from the Moderate Resolution Imaging Spectroradiometer (MODIS), which is currently available on a 1˚x1˚ grid from February 2000 to August 2019. MODIS anomalies are based on a long-term climatology for the period 2001-2018.

Net surface shortwave radiation (Q_{sw}) data were taken from the National Aeronautic and Space Administration's (NASA) Cloud and the Earth's Radiant Energy System (CERES). Specifically, Q_{sw} results are based on the CERES Energy Balanced and Filled (EBAF) dataset[1], which is available as monthly mean values from March 2000 to January 2019. CERES-EBAF Q_{sw} anomalies are based on a long-term climatology for the period 2001-2018.

Additional AGCM experiments

Using GFDL-AM2.1, we conduct a fourth and fifth set of experiments in which we decompose the total tropical sea surface temperature (SST)-forcing into the tropical Indian and tropical Pacific Ocean component. For these runs, we use 150˚E as the separating point between the two experiments (dashed purple line, Fig. 4). Each ensemble consists of 20 ensemble members forced under the same initial conditions and greenhouse gas concentrations as the total tropical

SST-only runs. Results for the tropical Pacific SST-only ensemble mean are shown in Supplementary Figure 4. Indian Ocean results showed no skill at reproducing observations.

Decomposition of the net surface heat flux in Blob 2.0 region

It is useful to decompose the contribution of the net surface heat flux, Q_{net}, to the temperature tendency shown in Fig. 3a, such that:

$$Q_{\mathrm{net}} = Q_{\mathrm{lh}} + Q_{\mathrm{sh}} + Q_{\mathrm{sw}} + Q_{\mathrm{lw}} \qquad (1)$$

where Q_{net} is the sum of the latent heat flux (Q_{lh}), the sensible heat flux (Q_{sh}), the net surface shortwave radiation (Q_{sw}), and the net surface longwave radiation (Q_{lw}). Given that the summertime mixed layer depth, h, in the Blob 2.0 region is very thin relative to other seasons (Fig. 3b), it is important to further assess the modulation of the SST time tendency by perturbations in the mixed layer depth itself[2]. For example, separating Q_{net} divided by h into a mean component and perturbation component, Taylor expanding, and linearizing yields[3]:

$$\frac{1}{\rho Cp}\left(\frac{Q_{\mathrm{net}}}{h}\right)' \approx \frac{1}{\rho Cp}\left(\frac{Q'_{\mathrm{net}}}{\bar{h}} - \frac{\bar{Q}_{\mathrm{net}}h'}{\bar{h}^2}\right) \qquad (2)$$

where the primes denote time anomalies and overbars represent the monthly climatology. The term on the left-hand side (LHS) of Eq. (2) represents the full influence of a time-varying heat flux and a time-varying mixed layer depth on the temperature tendency (hereafter referred to as Full), the first term on the right-hand side (RHS) represents changes in the mixed layer temperature due to anomalies of the surface heat flux (hereafter referred to as Flux), while the second term represents changes in the mixed layer temperature due to anomalies of mixed layer thickness acting on the mean surface heat flux (hereafter referred to as MLD)[4]. The RHS and LHS are only approximately equal due to neglecting higher order and non-linear terms in the Taylor expansion.

We can use Q_{lh} as an example to gain some physical intuition of what to expect from the Flux and MLD terms. In this case, the Flux term is related to SST anomalies that result from Q_{lh} anomalies mixing over the mean mixed layer depth (e.g., reducing wind speeds generates downward Q_{lh} anomalies and positive SST anomalies). In contrast, the MLD term is related to SST anomalies that result from mixed layer depth anomalies that then alter the depth over which mean surface heat fluxes are mixed. For example, $\bar{Q}_{\mathrm{lh}}$ is always a cooling term in the Blob 2.0 region (not shown). Therefore, seasons with anomalously thin mixed layers (i.e., large negative h') would allow $\bar{Q}_{\mathrm{lh}}$ to be mixed over a smaller volume, producing negative SST anomalies.

Figure S2 shows Eq. (2) applied to each heat flux component outlined in Eq. (1). We use monthly mean NCEP Reanalysis 2 (NCEP2) data to estimate Q_{net}, Q_{lh}, and Q_{sh} (see main Methods for more details on this data). However, a careful comparison of Q_{sw} maps from NCEP2 with MODIS low-cloud fraction anomalies shows significant inconsistencies, and does not provide enough confidence to use NCEP2 for the surface radiative fluxes. There are no other reliable direct observations of Q_{sw} and Q_{lw} that are currently available for June-August (JJA) 2019 in our region of interest. Therefore, we instead utilize the linear relationship between CERES-EBAF surface Q_{sw} and Q_{lw} anomalies and MODIS low-cloud fraction anomalies averaged during JJA from 2001-2018 at each grid point in the Blob 2.0 region (Supplementary Figure 5). By multiplying the respective regression coefficients with the JJA MODIS low-cloud fraction anomaly averaged in the Blob 2.0 region each year, we can estimate the surface radiative fluxes. Finally, note that the MLD values of the Q_{sh} are small because $\bar{Q}_{\mathrm{sh}}$ (-0.18 W m^{-2}) << $\bar{h}$ (26.23 m).

Low-cloud radiative effect vs. wind speed-induced latent heat flux in Blob 2.0 region

As discussed in the main text, satellite-derived observations show a reduction in low-cloud fraction in the Blob 2.0 region (shading, Fig. 6), which would tend to correspond to an increase in

surface downwelling solar radiation and, over a dark ocean, overall Q_{sw}. In order to estimate the radiative effect induced by these low-cloud anomalies, we again utilize the linear relationship between JJA averaged CERES-EBAF surface Q_{sw} and MODIS cloud data (Supplementary Figure 5a). The regression coefficient between these two variables is –1.19 W m^{-2} %$^{-1}$ based on a linear least-squares fit. Multiplying this coefficient by the JJA 2019 low-cloud fraction anomalies averaged in the Blob 2.0 region (–9.58%) provides an estimate of the low-cloud-induced increase in surface Q_{sw}, which equals 11.37 W m^{-2}.

It is interesting to compare the magnitude of the low-cloud radiative effect with the wind speed-induced downward latent heat flux[5,6] in the Blob 2.0 region. This allows us to assess the relative importance of these budget terms to the overall intensity of the Blob 2.0 SSTs. We calculate the wind speed-induced latent heat flux (lh_w) as:

$$lh_{\mathrm{w}} = \bar{Q}_{\mathrm{lh}} \frac{w'}{\bar{w}} \quad (3)$$

Where Q_{lh} the total latent heat flux and w is the scalar wind speed. Overbars denote the JJA climatology, and primes denote deviations from this mean. Using scalar wind speed data from NCEP Reanalysis 1 and latent heat flux data from NCEP Reanalysis 2, Eq. (3) estimates a wind speed-induced downward latent heat flux of 5.09 W m^{-2} during JJA 2019 in the Blob 2.0 region. This value compares well to the JJA 2019 total latent heat flux averaged in the Blob 2.0 region (5.43 W m^{-2}).

Supplementary References

1. Kato, S. *et al.* Surface irradiances of edition 4.0 Clouds and the Earth's Radiant Energy System (CERES) Energy Balanced and Filled (EBAF) data product. *J. Clim.* **31**, 4501–4527 (2018).
2. Alexander, M. Extratropical Air-Sea Interaction, Sea Surface Temperature Variability, and the Pacific Decadal Oscillation. in *Climate Dynamics: Why Does Climate Vary* 123–148 (2013). doi:10.1029/2008GM000794
3. Alexander, M. A. & Penland, C. Variability in a mixed layer ocean model driven by stochastic atmospheric forcing. *J. Clim.* **9**, 2424–2442 (1996).
4. Foltz, G. R., McPhaden, M. J. & Lumpkin, R. A strong atlantic meridional mode event in 2009: The role of mixed: Layer dynamics. *J. Clim.* **25**, 363–380 (2012).
5. Du, Y., Xie, S.-P., Huang, G. & Hu, K. Role of air-sea interaction in the long persistence of El Niño-induced north Indian Ocean warming. *J. Clim.* **22**, 2023–2038 (2009).
6. Amaya, D. J., DeFlorio, M. J., Miller, A. J. & Xie, S. P. WES feedback and the Atlantic Meridional Mode: observations and CMIP5 comparisons. *Clim. Dyn.* **49**, 1665–1679 (2017).

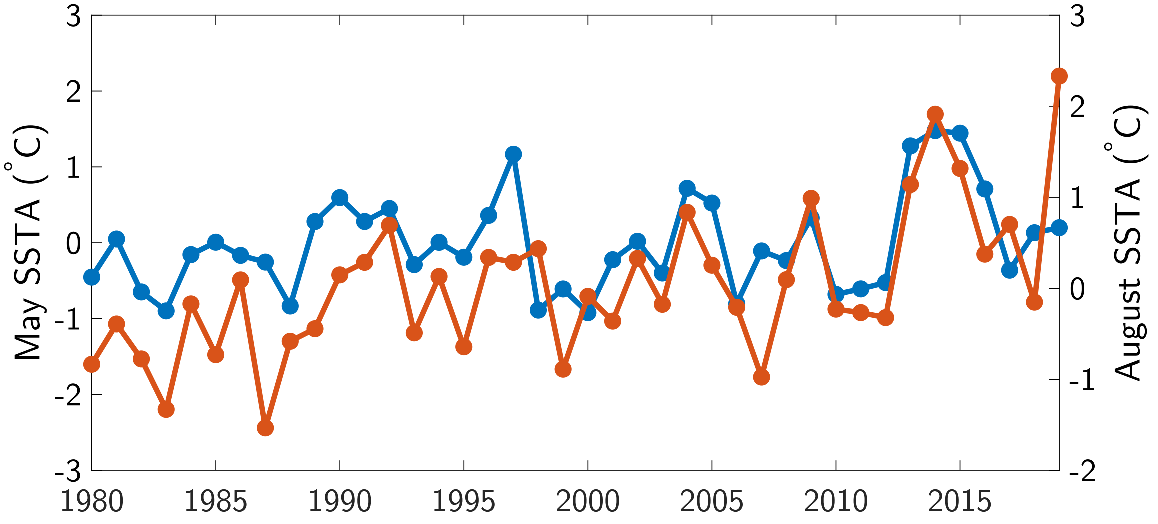

Supplementary Figure 1 May and August surface temperature anomalies. Five-meter ocean temperature anomalies (˚C) in May (blue) and August (red) in Global Ocean Data Assimilation System (GODAS) area-weighted averaged in black box shown in Fig. 1a.

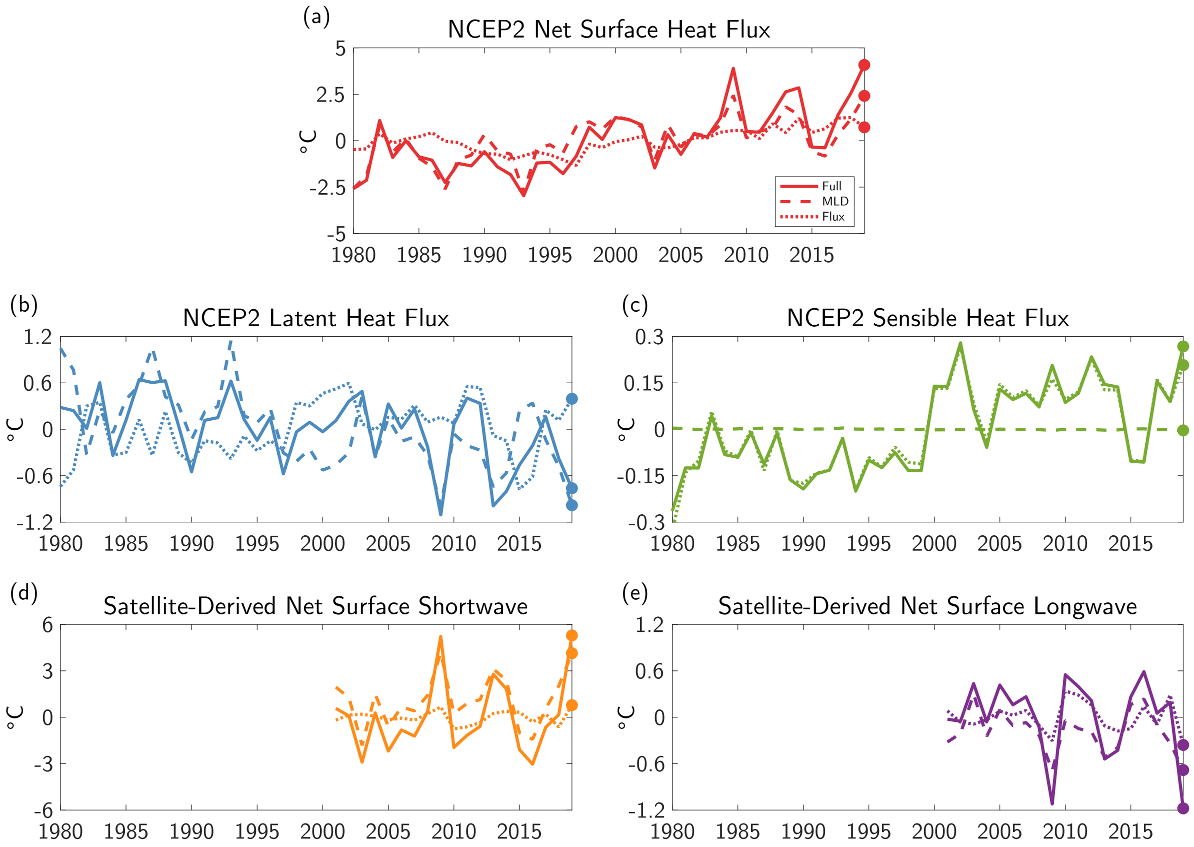

Supplementary Figure 2 Surface heat flux budget term components. Terms from Eq. (1) contributing to the August minus May temperature change depicted in Fig. 3a Using Eq. (2), each flux term has been separated into contributions from a time-varying mixed layer depth (MLD; dashed), a time-varying heat flux (Flux; dotted), and the total time-varying heat flux over mixed layer depth term (Full; solid). Note each term has been multiplied by three months to convert units to degrees Celsius.

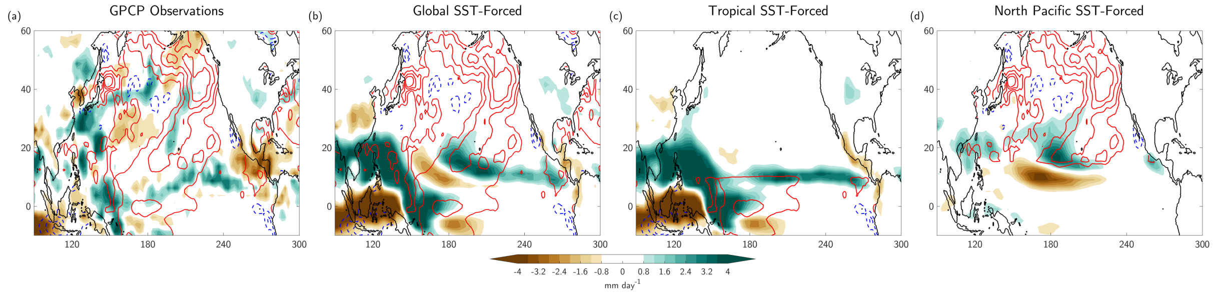

Supplementary Figure 3 Precipitation anomalies during summer 2019. Precipitation anomalies (shading; mm day^{-1}) with underlying sea surface temperature anomalies (SSTAs; red/blue contours; ˚C) averaged for June-August 2019. Results shown for (a) Observations and the ensemble means of: (b) global SST-forced, (c) tropical SST-forced, and (d) North Pacific SST-forced atmospheric model simulations. For (b)-(d), SSTAs only shown in forcing domain. Contour interval is 0.5˚C, with a maximum value of 2˚C.

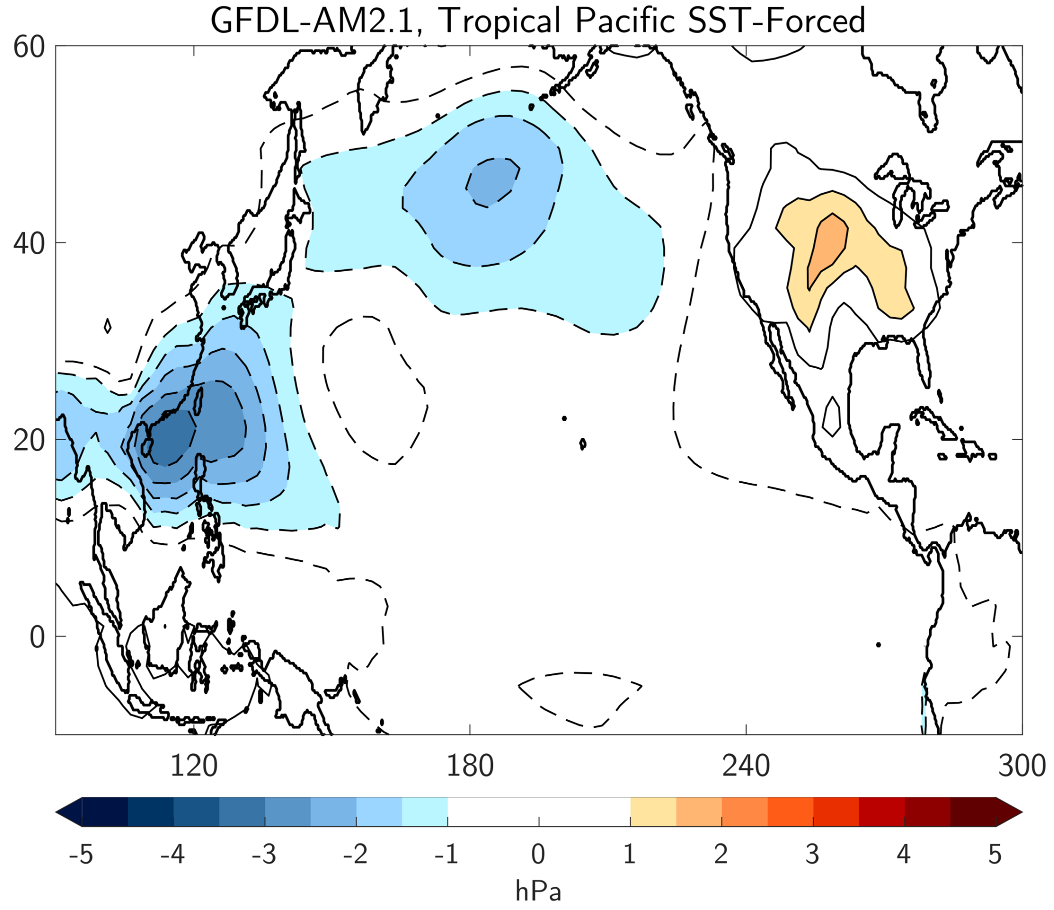

Supplementary Figure 4 Circulation anomalies for tropical Pacific-only simulation. Ensemble mean sea level pressure anomalies (SLPAs; hPa) for tropical Pacific-only atmospheric model simulation, averaged for June-August 2019.

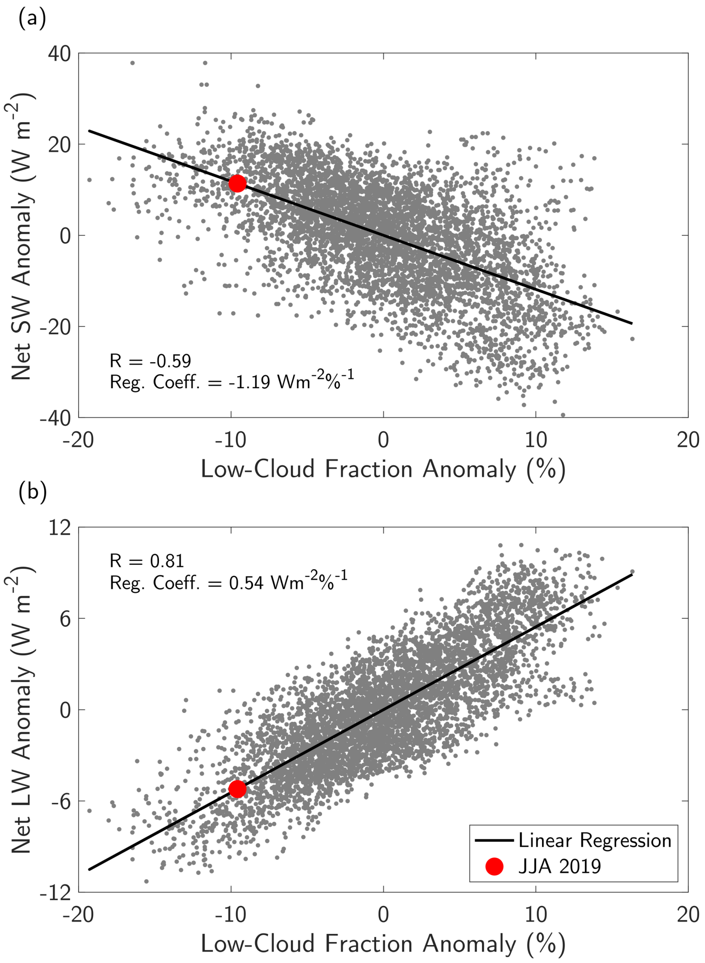

Supplementary Figure 5 Satellite-derived low-cloud fraction and radiation anomalies. Scatter of satellite-derived low-cloud fraction (%; x-axes) and surface net radiation anomalies (W m^{-2}; y-axes). (a) Low-cloud fraction anomalies versus net shortwave (SW) anomalies. (b) Low-cloud fraction anomalies versus net longwave radiation (LW) anomalies. Data based on June-August (JJA) average for the period 2001-2018 and are shown at each grid point and time step in the Blob 2.0 region (Fig. 1a black box). Black line represents line of best fit based on linear least-squared regression. Red dot is JJA 2019 average. Its position along x-axis represents the low-cloud fraction anomaly averaged in the Blob 2.0 region. Its position along y-axis represents the SW and LW values estimated by multiplying the low-cloud fraction in the Blob 2.0 region by the respective regression coefficients.
